# Effects of plant density on the aboveground dry matter and radiation-use efficiency of field corn

**DOI:** 10.1371/journal.pone.0277547

**Published:** 2022-11-10

**Authors:** Yi-Chin Li, Hung-Yu Dai, Hungyen Chen

**Affiliations:** 1 Department of Agronomy, National Taiwan University, Taipei, Taiwan; 2 Taiwan Agricultural Research Institute, Council of Agriculture, Executive Yuan, Taichung, Taiwan; Hunan Agricultural University, CHINA

## Abstract

The amount of solar radiation intercepted by the plant canopy drives crop plant photosynthesis and the formation and development of plant organs. Radiation-use efficiency (RUE) is an index used to quantify the relationship between solar radiation and biomass, and crop yield can be increased by increasing RUE. The main goals of this study were to initially investigate the effects of plant densities on the aboveground dry matter of corn, and subsequently examine the effects of plant densities on RUE and leaf area index (LAI), and the effects of LAI on RUE. Finally, we provide a comparative assessment of the approaches used to determine RUE. Analyses were conducted using growth and meteorological data obtained for two field corn varieties (TNG1 and TNG7) grown under four different plant density conditions in central Taiwan in 2017. The RUE values obtained in this study were primarily estimated from the slope of the linear relationship between aboveground dry matter measured at periodic harvests and the corresponding cumulative intercepted photosynthetically active radiation up to the time of harvest. TNG1 and TNG7 with a row spacing of 37.5 cm × 20 cm had the largest amounts of aboveground dry matter and highest RUE values of 4.41 and 4.55 g MJ^-1^, respectively. We established that the higher the plant density, the higher were the values obtained for RUE and LAI. We also compared the different methods of estimating RUE and make recommendations in this regard. Our findings in this study will enable farmers to gain information on the dynamics of crop yield variation at an early stage of growth, and also provide reference values that can be incorporated in future crop yield models.

## Introduction

It has been estimated that for each 1 C increase in average global temperature, maize yields will be reduced by an average of 7.4% [1, 2]. In this regard, the findings of a meta-analysis conducted by Stanhill and Cohen [3] indicated that in the past 50 years, global solar radiation has declined by an average of 2.7% per decade, and that solar radiation has generally declined in many parts of the world [4]. Low solar radiation will have the effect of weakening internodes at the base of corn, leading to the growth of elongated fragile stems, and thereby increasing the probability of lodging and adversely affecting maize yields [5]. Reduced solar radiation can adversely affect the filling of corn kernels, resulting in lower yields [6]. Under the influence of global climate change, the probability of extreme weather increases. High temperature and concentrated precipitation will affect the growth and development of maize and inevitably lead to reductions in yield [7, 8]. Therefore, there is an urgent need for information on maize growth and environmental weather [9, 10].

In meeting future production demands, adapting crop growth to the prevailing environmental conditions will be an important innovation that can contribute to increasing total biomass and grain yield [11–14]. High maize yields tend to be obtained under conditions of relatively low temperature and high solar radiation [15]. Theoretically, crop yields can therefore be improved by enhancing radiation-use efficiency (RUE) [16, 17], a parameter used to quantify the relationship between solar radiation and biomass production, which is the input basis for growth and yield models [18, 19]. Several studies with regards to climate change and RUE have been reported recently [20–22]. A number of different methods for calculating RUE have been described in the literature. Sinclair and Muchow [23], for example, mention that RUE can be calculated by dividing the biomass difference between two consecutive harvests by the corresponding amount of intercepted radiation. Generally, however, RUE is defined as the ratio of dry matter produced per unit of radiant energy used in its production [24]. Estimates of RUE are also dependent on whether radiation is measured as intercepted total solar radiation or as absorbed or intercepted photosynthetically active radiation (APAR or IPAR, respectively) [23, 25, 26]. The maximum RUE of maize in vegetative growth period has been estimated at 1.6 g MJ^-1^, whereas Loomis and Amthor [27] estimated potential maize RUE at 4.9 g of the total biomass production per unit APAR, with APAR being calculated as incident photosynthetically active radiation (PAR) minus transmitted and reflected PAR. Hatfield [28] found that the values for RUE showed a linear relationship between biomass and IPAR for the entire growing season, the latter of which can be estimated from Beer’s Law [29, 30]. The most common method used to calculate RUE uses the slope of the relationship between dry matter production and cumulative intercepted photosynthetically active radiation (IPAR), as has been described in numerous studies [31–33]. Sinclair and Muchow [23] stated that in those cases in which a conversion technique is not explicitly described in a paper, it is generally assumed that the ratio of PAR to total radiation in a direct solar beam is 0.5 [34]. Monteith [35] also suggests an average value of 0.5 is probably appropriate in both tropical and temperate latitudes. In the present study, we assume that leaves completely intercept PAR as IPAR in the absence of any radiative loss or leaf area index (LAI) effects.

Incident solar radiation striking the canopy is absorbed, transmitted, or reflected, the varying extents of which are dependent on the angle of incidence of the solar rays and the quantitative and qualitative characteristics of the canopy [36]. In this context, numerous research reports have indicated that higher yields per unit area can be obtained by increasing the planting density of field corn. Yu et al. [10] shaded maize plots with different degrees of shading, simulating different degrees of solar radiation in different corn planting regions, whereas Sarmento et al. [19] evaluated RUE for the accumulated biomass and grain yield based on the sowing dates and plant densities in Brazil in 2013. Their results consistently show that with sufficient solar radiation, maize yields can be increased in response to a higher planting density, which can be attributed to the fact that increasing the density of plants has the effect of altering the distribution and angle of leaves, thereby increasing the interception of incident solar radiation. LAI is the sum of all leaf areas per unit area of land, and changes in the distribution and size of leaf area are closely related to crop energy transfer and dry matter accumulation [37]. However, the degree of intra-specific competition caused by increased plant density can cause a reduction in the plant parts of interest, such as the grain [38, 39]. Therefore, studies using populations of differing densities are needed to determine the optimal utilization of solar radiation, water, and nutrients.

The main goals of this study were to initially investigate the effects of plant density on aboveground maize dry matter, and subsequently examine the effects of plant densities on RUE and LAI, and the effects of LAI on RUE. Finally, we undertook a comparative assessment of the approaches used to determine RUE. As plant materials, we used the field corn varieties Tainung No.1 (TNG1) and Tainung No.7 (TNG7), which were planted at the Taiwan Agricultural Research Institute Council of Agriculture Executive Yuan. The plants were subjected to four different density treatments and on the basis of our examination of the effects of plant densities on RUE, we recommend the most suitable plant variety and planting density. We anticipate that our finding will enable farmers to gain information on the dynamics of crop yield variation at an early stage of growth, and also provide reference values that could be incorporated in future crop yield models.

## Materials and methods

### Field cultivation of TNG1 and TNG7

Data were obtained from experimental field No. 30 of the Taiwan Agricultural Research Institute Council of Agriculture Executive Yuan. The experimental design was a randomized complete block design with four replications. The field corn varieties assessed were Tainung No.1 (TNG1) and Tainung No.7 (TNG7). These were subjected to four different treatments with row space of 75.0 cm × 20 cm, 37.5 cm × 40 cm, 37.5 cm × 27 cm, and 37.5 cm × 20 cm, which are equivalent to 7 (D1), 7 (D2), 10 (D3), and 14 (D4) plants per square meter unit area (8 treatments in total). We accordingly used a total of 32 plots (8 treatments × 4 replicates), each of which was 5.5 m long and 5.25 m wide, and cultivation was performed without ridging. Planting commenced on September 27, 2017, in soils to which a base fertilizer (N:P:K = 12:18:12) had been added 2 days previously at a rate of 45 kg per 0.1 ha. As pre-emergence herbicides for weed management, we applied a 200-fold dilation of 1 kg powdered Atrazine and a 200-fold dilution of 1250 mL of Pendimethalin on September 29, 2017. Plots were irrigated on October 5, October 27, November 9, and December 22, 2017. For pest control we applied a 1000-fold dilution of 700 mL of Bifenthrin and an 850-fold dilution of 840 g Carbaryl on October 12, 2017, and insecticides (a 2000-fold dilution of Tebufenozide and an 800-fold dilution of Profenofos) were sprayed on October 30, 2017. A top dressing of fertilizer (N:P:K = 20:5:10) was applied to the soil at a rate of 45 kg per 0.1 ha. From the 3^rd^-leaf stage, three maize plants were dug up from each plot at 10-day interval to investigate the leaf age, leaf area, aboveground dry matter, and root dry matter. We also obtained samples at the silking stage (R1) and at harvest performed at the stage of physiological maturity (R6) (Table 1) [40], and examined a selected range of agronomic traits, including yield, the dry matter of different plant parts, and the number of spikes.

**Table 1:** Growth and development stages in corn.

Vegetative Stages		Reproductive Stages	
VE	Emergence	R1	Silking—silks visible outside the husks
V1	First leaf collar	R2	Blister—kernels are white and resemble a blister in shape
V2	Second leaf collar	R3	Milk—kernels are yellow on the outside with a milky inner fluid
V3	Third leaf collar	R4	Dough—milky inner fluid thickens to a pasty consistency
V(n)	nth leaf collars visible	R5	Dent—nearly all kernels are denting
VT	Tasseling—last branch of tassel is completely visible	R6	Physiological maturity—the black abscission layer has formed

The TNG1 variety, which is mainly used as animal feed, was bred by the Taiwan Agricultural Research Institute Council of Agriculture Executive Yuan in 1987. It is a single-hybrid variety with tall plants, broad leaves, dent-shaped grains, high yield, good disease resistance, and excellent traits such as being suitable for mechanical harvesting. Depending on the cultivation environment, the growth period ranges from 105 to 130 days [41]. TNG7 was similarly bred by the Taiwan Agricultural Research Institute Council of Agriculture Executive Yuan in 2017, with the aim of producing a variety with traits superior to those obtained in TNG1. The grain is dent-shaped, and the plants have excellent traits such as medium-early maturity, high yield, and rust resistance. The growth period is similar to that of TNG1, ranging from 105 to 135 days, again depending on the cultivation environment [42].

### Climatic data

Data of global solar radiation were obtained from the weather station of the Taiwan Agricultural Research Institute Council of Agriculture Executive Yuan (station code G2F820), located in Wanfeng Village, Wufeng District, Taichung City for 13 months from January 1, 2017, to January 31, 2018.

### Data arrangement

TNG1 and TNG7 plants were sampled for aboveground dry matter determinations at 10-day intervals from the 3^rd^-leaf stage commencing from September 27, 2017, and with the final samples being collected on February 22, 2018, when the plants had reached physiological maturity. In total, plants were harvested for investigations at 12 time points throughout the entire growth period (Table 2). The original data unit is the average dry matter of each plant under the four different treatments. The expected plant numbers per square meter under four different treatments were 7, 7, 10 and 14, respectively. In order to convert the data to dry matter weight per square meter, the original data were multiplied by the expected number of plants per square meter under the four different treatments. The aboveground dry matter includes that of the leaf blades, leaf sheaths, stems, and ears.

**Table 2:** Investigation date of TNG1 and TNG7.

Date	Days
2017/09/27	0
2017/10/18	21
2017/10/27	30
2017/11/06	40
2017/11/16	50
2017/11/27	61
2017/12/07	71
2017/12/18	82
2017/12/27	91
2018/01/08	103
2018/01/18	113
2018/01/29	124
2018/02/22	148 (Physiological maturity stage, R6)

The original meteorological data represent daily records of the assessed variables. In order to correspond with the field survey data, all meteorological data were accumulated and sorted according to the survey date. Given that meteorological data for the period between February 1 and February 28, 2018, were unavailable from Taiwan Agricultural Research Institute Council of Agriculture Executive Yuan, and that the final harvests of TNG1 and TNG7 were on February 22, 2018, we obtained meteorological data (albeit incomplete) for this period from the Taichung weather station of the NAGR Monitor of the Central Weather Bureau, (station code G2F82) Therefore, for the relevant solar radiation for February 2018, we used the solar radiation data of the gridded satellite retrieval daily data provided by the Taiwan Climate Change Projection Information and Adaptation Knowledge Platform (TCCIP). The longitude and latitude of the Taiwan Agricultural Research Institute Council of Agriculture Executive Yuan are 120.68 and 24.03, respectively, whereas those of the closest meteorological station are 120.65 and 24, respectively, which are used for the convenience of subsequent analysis. The central average temperature of the gridded observational day data provided by TCCIP was taken as the average temperature for February 2018.

### Radiation-use efficiency

RUE can be calculated using different measurement data, although for the purposes of the present study, we calculated RUE as the ratio of aboveground total dry weight to the IPAR (MJ m^-2^) across the entire growing season [21]. On the basis of the findings of previous studies, we assumed that PAR was 50% of the global solar radiation [23, 34, 35]. Moreover, we assumed that leaves completely intercept PAR as IPAR in the absence of any radiative loss and leaf area index (LAI) effects. RUE relates to the aboveground dry matter produced per unit of intercepted PAR, the values of which (g MJ^-1^) were estimated based on the relationship between the accumulation of aboveground dry matter (g m^-2^) and IPAR (MJ m^-2^) as follows:

$$IPAR\;\left(MJ\;m^{-2}\right)=0.5\times Global\;solar\;radiation\;\left(MJ\;m^{-2}\right).$$

RUE was calculated as the slope of simple linear regression using accumulative dry matter as dependent variable and IPAR as independent variable:

$$Aboveground\;dry\;matter\;\left(g\;m^{-2}\right)=Intercept\;term\;\left(g\;m^{-2}\right)+RUE\;\left(g\;{MJ}^{-1}\right)\times IPAR\;\left(MJ\;m^{-2}\right).$$

### Leaf area index

The leaf area index is defined as the ratio of the total leaf area of crops to the land area on a fixed land area (unit area) and is calculated using the following formula:

$$LAI=\frac{Leaf\;area\;\left({cm}^{2}/plant\right)*Number\;of\;plants\;per\;unit\;area\;(plants)}{100(cm)*100\;(cm)}$$

### Statistical analysis

Changes in the aboveground dry matter of the two different field corn varieties with growth period under the four different treatments, separately, were analyzed using an analysis of variance (ANOVA). To test the significance of the effects of planting density, the following one-way ANOVA model for the variable X_*ij*_ at planting density *i* for the *j*th plant was used:

$$X_{i,j}=Mean+{Planting\;density}_{i}+{Error}_{ij}$$

For multiple comparisons, least significant difference (LSD) tests were performed to determine whether there were significant differences between the four different treatments. We also assessed the relationship between accumulative aboveground dry matter and cumulative IPAR based on regression analysis. The data obtained for the aboveground dry weight of field corn after processing are expressed as mean values. The linear relationship between plant per unit area and RUE was also analyzed:

$$RUE\;\left(g\;{MJ}^{-1}\right)=Intercept\;term\;\left(g\;{MJ}^{-1}\right)+Slope\;\left(g\;{MJ}^{-1}\;{plant}^{-1}\;m^{2}\right)\times Plant\;per\;unit\;area\;\left({plant\;m}^{-2}\right).$$

All calculations and statistical analyses were performed using R 4.1.2 statistical software.

## Results

### Aboveground dry matter accumulation of TNG1 and TNG7 under four different density during the entire growth period

**Fig 1:**
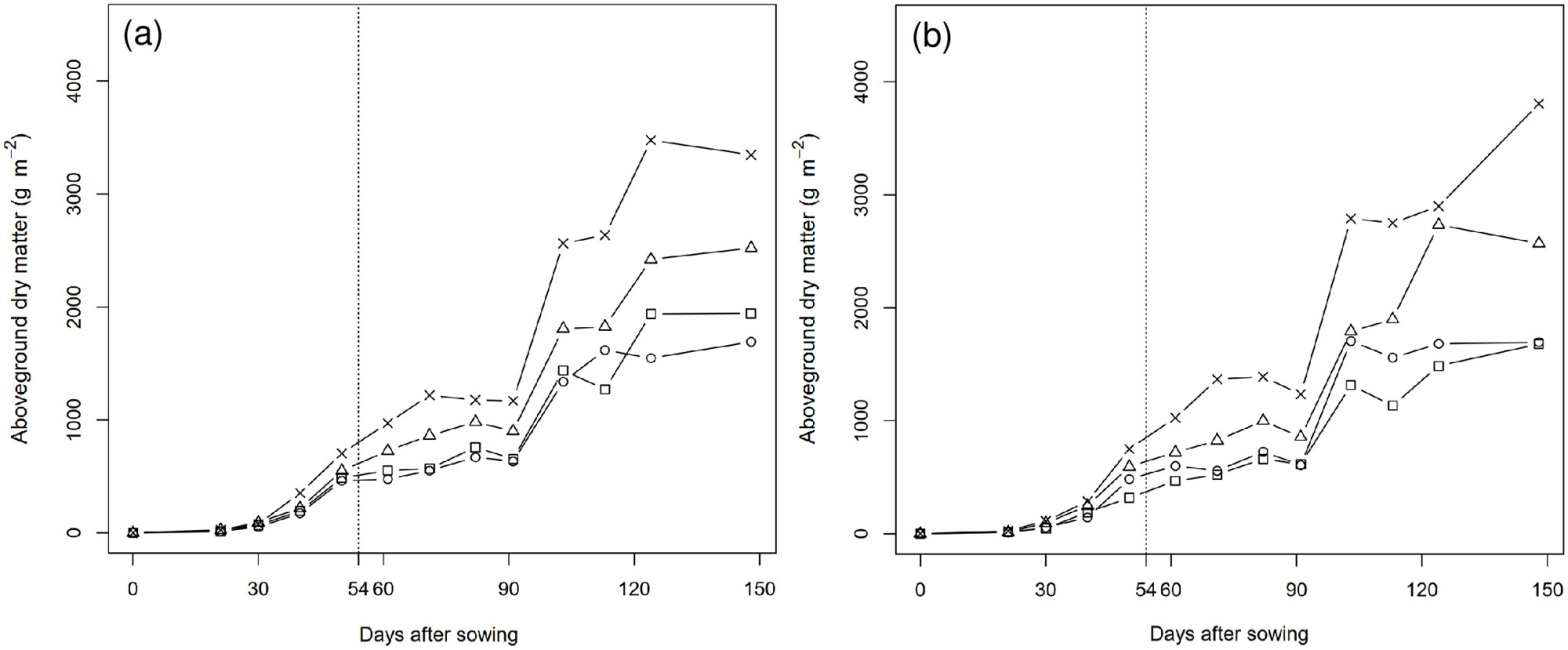
Changes in aboveground dry matter accumulation (g m^-2^) of TNG1 (a) and TNG7 (b) under four different treatments (cm^2^) during the whole growth period. Squares represent D1: 75.0 × 20, 7 plants m^-2^, circles represent D2: 37.5 × 40, 7 plants m^-2^, triangles represent D3: 37.5 × 27, 10 plants m^-2^ and crosses represent D4: 37.5 × 20, 14 plants m^-2^. The 54th day after sowing is silking stage, which is indicated by the dashed line.

Generally, we detected significant difference in the aboveground dry matter of the TNG1 and TNG7 varieties subjected to the four different density treatments using the ANOVA test, separately (Table 3). The silking stage commenced on day 54 after sowing, and the vegetative growth was vigorous. The aboveground dry matter per unit area of TNG1 and TNG7 were highest in treatment D4 at 3346.12 and 3806.13 g m^-2^, respectively, followed by the respective values of 2521.58 and 2569.92 g m^-2^ in treatment D3. Somewhat lower values were obtained in treatments D1 and D2, with respective values of 1944.08 and 1691.55 g m^-2^ for TNG1, and 1680.23 and 1695.98 g m^-2^ for TNG7 (Fig 1). We observed that dry matter increased with an increase in plant density, and detected no significant difference in dry matter per unit area under treatments D1 and D2, in which the number of plants per unit area was the same, (Table 3).

**Table 3:** The aboveground dry matter (g m^-2^) of TNG1 and TNG7 affected by four different treatments (cm^2^).

Variety	Treatment	Aboveground dry matter
TNG1*	D1: 75.0 × 20	1944.08^c1^
	D2: 37.5 × 40	1691.55^c1^
	D3: 37.5 × 27	2521.58^b1^
	D4: 37.5 × 20	3346.12^a1^
TNG7*	D1: 75.0 × 20	1680.23^c2^
	D2: 37.5 × 40	1695.98^c2^
	D3: 37.5 × 27	2569.92^b2^
	D4: 37.5 × 20	3806.13^a2^

* represents significant difference in the aboveground dry matter subjected to the four different density treatments using an ANOVA test (*p*-value < 0.001). Values of aboveground dry matter denoted by the same lower-case letter do not differ significantly at the 5% level, as determined by a least significant difference test.

### Effects of plant density on RUE and LAI

**Fig 2:**
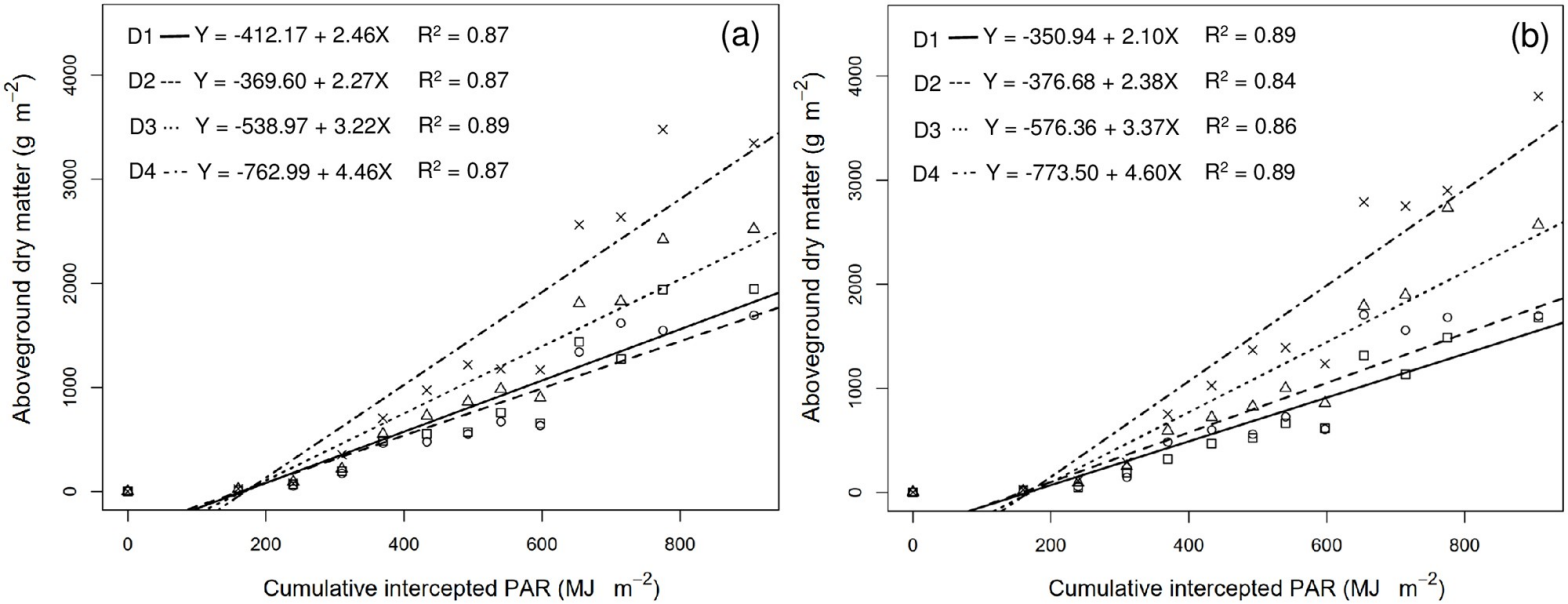
Relationship between the accumulation aboveground dry matter (g m^-2^) and cumulative intercepted photosynthetically active radiation (IPAR, MJ m^-2^) in TNG1 (a) and TNG7 (b) under four different treatments (cm^2^) during the whole growth period. Squares represent D1: 75.0 × 20, 7 plants m^-2^, circles represent D2: 37.5 × 40, 7 plants m^-2^, triangles represent D3: 37.5 × 27, 10 plants m^-2^ and crosses represent D4: 37.5 × 20, 14 plants m^-2^. Each RUE calculated as the slope of the simple linear regression line.

**Fig 3:**
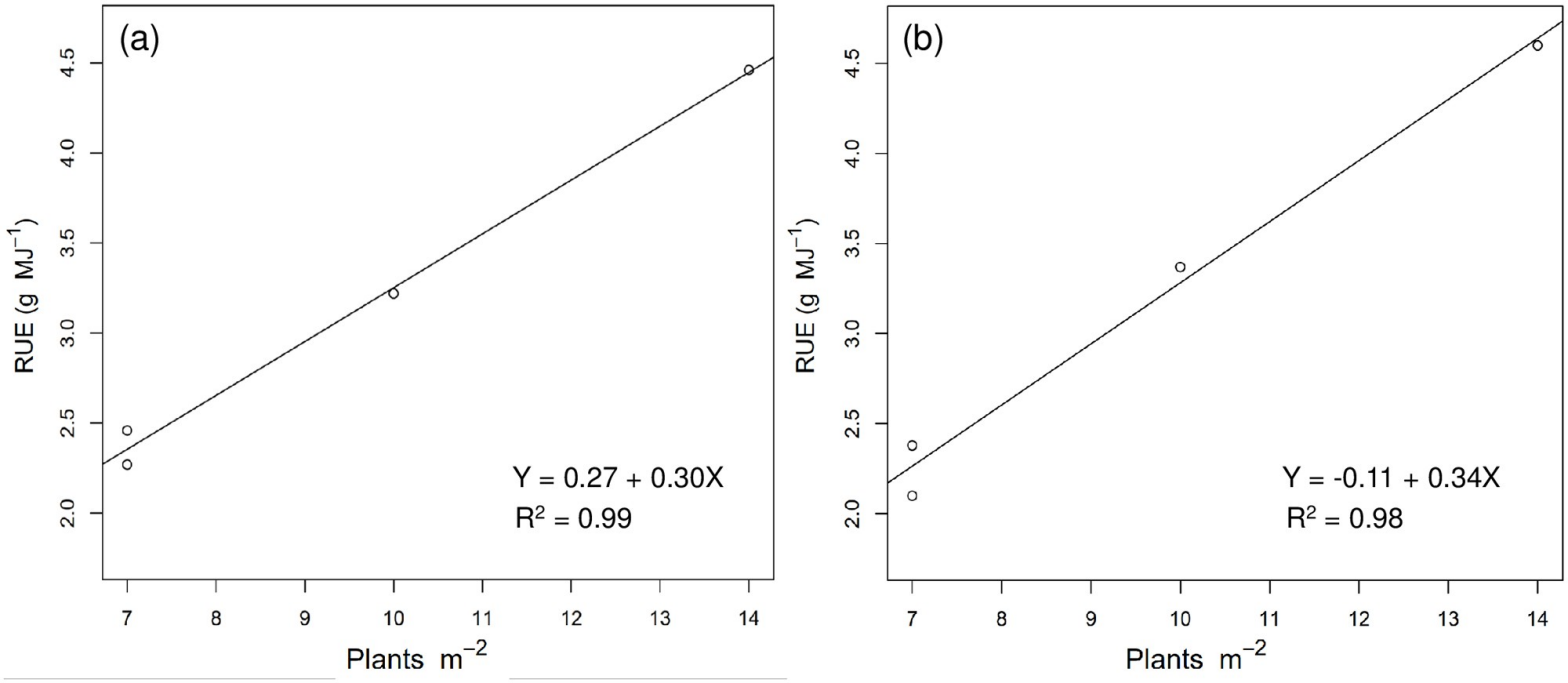
Relationship between the radiation use efficiency (RUE, g MJ^-1^) and plant density in TNG1 (a) and TNG7 (b). Circles represent plant density D1: 75.0 × 20, 7 plants m^-2^, D2: 37.5 × 40, 7 plants m^-2^, D3: 37.5 × 27, 10 plants m^-2^, and D4: 37.5 × 20, 14 plants m^-2^.

From September 27, 2017, to February 22, 2018, the end of physiological maturity was recorded for a total of 148 days. The accumulative global solar radiation during the entire growth period was 1813.43 MJ per unit area, and after conversion, we obtained a cumulative IPAR value of 906.72 MJ per unit area for this period. The slope of the relationship between aboveground dry matter and cumulative IPAR corresponds to RUE. The RUE value of 2.46 g MJ^-1^ IPAR obtained for TNG1 corn under treatment D1 indicates that the corn yielded 2.46 g of dry matter per megajoule of photosynthetically active radiation. RUE values under treatments D2, D3, and D4 were 2.27, 3.22, and 4.46 g MJ^-1^ IPAR, respectively, whereas for the TNG7 variety, we obtain values of 2.10, 2.38, 3.37, and 4.60 g MJ^-1^ IPAR in response to treatments D1, D2, D3, and D4, respectively (Fig 2 and Table 4). In the case of both TNG1 and TNG7, we detected a highly positive correlation between RUE and plant density under the four different treatments, with R^2^ values of 0.99 and 0.98, respectively, which reasonably describe the relationship between variables. Overall, we found that the higher the plant density per unit area, the higher was the RUE, and for both TNG1 and TNG7, we recorded the highest RUE in response to treatment D4 (Fig 3).

**Table 4:** Radiation use efficiency (RUE, g MJ^-1^) and maximum leaf area index (LAI) of four different treatments (cm^2^) for TNG1 and TNG7.

Variety	Treatment	RUE	LAI
TNG1	D1: 75.0 × 20	2.46	3.34
	D2: 37.5 × 40	2.27	3.04
	D3: 37.5 × 27	3.22	4.49
	D4: 37.5 × 20	4.46	6.02
TNG7	D1: 75.0 × 20	2.10	3.04
	D2: 37.5 × 40	2.38	3.52
	D3: 37.5 × 27	3.37	4.08
	D4: 37.5 × 20	4.60	5.95

**Fig 4:**
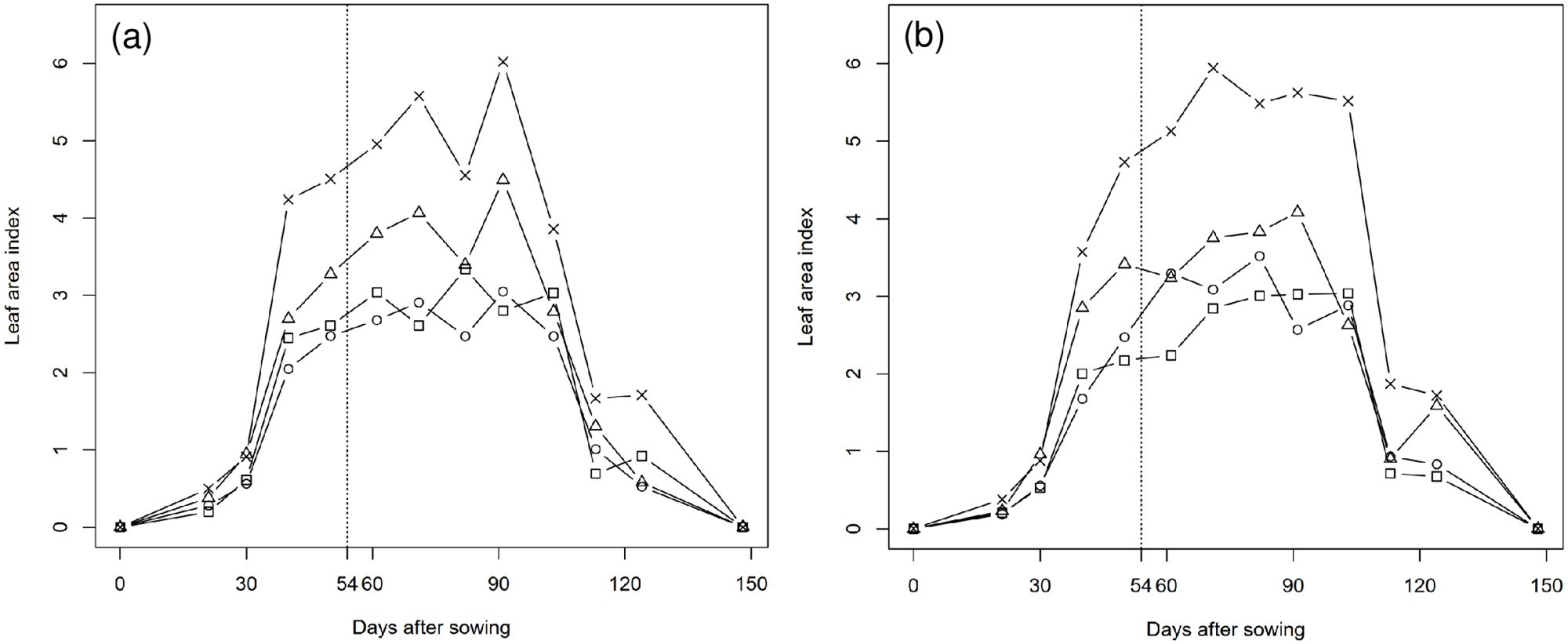
Change of leaf area index (LAI) during the whole growth period of TNG1 (a) and TNG7 (b) under four different treatments (cm^2^). Squares represent D1: 75.0 × 20, 7 plants m^-2^, circles represent D2: 37.5 × 40, 7 plants m^-2^, triangles represent D3: 37.5 × 27, 10 plants m^-2^ and crosses represent D4: 37.5 × 20, 14 plants m^-2^. The 54th day after sowing is silking stage, which is indicated by the dashed line.

The TNG1 and TNG7 varieties were characterized by vigorous vegetative growth prior to the silking stage, and during this period there was a rapid increase in the LAI of plants under each of the four different treatments. Conversely, we detected no significant change in LAI from the silking stage to the milk stage, and the leaf area per unit land decreased rapidly after the milk stage. For both TNG1 and TNG7, we recorded the highest LAI under treatment D4, with values of 6.02 and 5.95, respectively (Fig 4). Both RUE and LAI values increased with an increase in plant density from treatment D1 to D4 (Table 4).

## Discussion

### Aboveground dry matter accumulation of TNG1 and TNG7 under four different density during the entire growth period

In the present study, prior to the silking stage, we detected no significant difference between TNG1 and TNG7 with respect to dry matter accumulation under the four different treatments. The post-silking stage was characterized by continued grain filling, along with a rapid increase and accumulation of aboveground dry matter (Fig 1). In this regard, Chen et al. [43] mention that the fresh weight yield reached a peak at 7 to 14 days after silking and subsequently decreased, although after the dough stage the biomass continued to accumulate. The optimum harvest period for field corn is the stage of physiological maturity, during which, the aboveground dry matter reaches a maximum. The economic yield of field corn corresponds to the dry matter of grain, and accordingly, the ideal time to harvest corn is when the accumulated dry matter reaches a maximum. This relationship can be incorporated in the establishment of crop models, and can aid in predicting the harvest period and contribute to planning effective field work schedules for different crops and varieties.

In this study, we subjected the TNG1 and TNG7 varieties to four different density treatments, among which, treatment D4 proved conducive to yielding the highest values for aboveground dry matter per unit area. Treatment D3 was identified as the second most effective treatment in this regard, with treatments D1 and D2 being found to be the least effective. We observed that the dry matter of the two corn varieties increased with an increase in plant density, and that dense planting was associated with higher values of dry matter per unit area. For treatments D1 and D2, in which the number of plants per unit area was the same, we detected no significant difference in dry matter per unit area (Fig 1 and Table 3). Similarly, Farnham [44] showed that under the same planting density per unit area, there were no significant differences in biomass between different row spacing treatments. Widdicombe and Thelen [45], who evaluated the effects of row spacing and plant density on maize grain yield in the northern Corn Belt, found that plots with the highest plant density in the study (90,000 plants per hectare) also had the highest maize grain yield. Chen and Lee [46] showed that regardless of spring and autumn cultivation, the maximum yield was obtained at a row spacing of 75 cm × 20 cm. The findings of these studies accordingly highlight that an appropriate density of cultivation can contribute to increasing field corn seed production. Yu et al. [10] mention that field maize Ming-Fung No. 3 can achieve higher yield with row spacings of 75 cm × 15 cm and 75 cm × 25 cm, and that high-density planting is an effective approach for increasing maize yields in modern maize production. However, the minimum row spacing treatment of 30 cm × 15 cm was found to result in severe plant disease and pest infestation, and even the yield was significantly reduced due to the high planting density. Sangoi [47] also mentions that an increase in the number of individuals per area beyond the optimum plant density will contribute to delayed ear differentiation and non-synchronous flowering, thereby resulting in reduced yields. Given that TNG1 and TNG7 are characterized by a range favorable plant traits, such as robustness, heat resistance, drought resistance, and disease resistance, increasing planting density will also increase the accumulation of aboveground dry matter.

### Effects of plant density on RUE and LAI

Dry matter production is related to light interception and to RUE. RUE represents the aggregated response of the crop to a multitude of factors that affect photosynthesis and respiration during the entire growing season or a certain part of the growing period [33, 48, 49]. On the basis of the findings of previous studies, it has been established that RUE can be used to explain the patterns of aboveground dry matter. In the present study, we estimated RUE from the slope of the linear relationship between aboveground dry matter and the cumulative IPAR. The highest RUE values recorded for TNG1 and TNG7 (4.46 g and 4.60 g MJ^-1^, respectively) were obtained in treatment D4, which indicates that these varieties can respectively produce 4.46 and 4.60 grams of dry matter per megajoule of IPAR. The second-highest values were obtained in response to treatment D3, whereas treatments D1 and D2 yielded the lowest values (Fig 2). In this regard, Muchow [50] showed that the maximum RUE value for maize (1.59 g MJ^-1^) was obtained during the vegetative growth period and that values decreased rapidly after the silking stage. In contrast, Tewes and Schellberg [51] obtained maximum RUE values for maize ranging from 4.05 to 4.65 g MJ^-1^, and found that biomass was still increasing when the final measurements taken, which is consistent with the findings of Lindquist et al. [52] and the results obtained in the present study. Furthermore, Sinclair and Muchow (1999) [23] reported maximum RUE values for maize ranging from 0.66 to 4.8 g MJ^-1^. Thus, the RUE values obtained in the present study are all within a reasonable range, and accordingly have reference value. The higher the number of plants per unit area (i.e., planting density), the greater is the amount of light that can be intercepted, thereby giving rise to a higher RUE. Furthermore, we detected a highly linear relationship between RUE and planting density (Fig 3). The higher the planting density, the higher is the interception of solar radiation and light. Our findings in this regard are consisted with those reported previously; for example, Sarmento et al. [19] obtained maximum RUE values for corn ranging from 3.71 to 4.09 g MJ^-1^, and found that higher RUE vales were obtained at higher planting densities. Similarly, Andrade et al. [53] and Lee [54] showed that high plant density treatments resulted in higher yields, which could be attributed to an increase in the light intercepted by the maize canopy. For maize, the interception of radiation is correlated with plant density, with a high plant density facilitating a greater interception of photosynthetic radiation and thus higher grain yields [55].

LAI is indicative of the photosynthetic capacity of crop communities, and is often used as an indicator of plant growth, as well as in evaluating assimilation and evapotranspiration rates in plant physiology studies. LAI summarizes the factors affecting the structures of plant canopies, which are influenced by interactions between plant genotype and environmental factors, and is accordingly suitable for the study of dry matter production [37, 56, 57]. Leaves are the main parts of plants that interface with the surrounding atmosphere, and LAI is important for understanding crop development and growth, and serves as a key indicator for evaluating mass and energy exchange. LAI reflects the actual plant state and production potential, and changes in productivity and growth are closely associated with the amount of intercepted radiation [58–60]. In the present study, we sought to analyze the dynamic changes in LAI during the entire growth period of field corn. Prior to the silking stage, TNG1 and TNG7 showed vigorous vegetative growth marked by an increase in leaf area per unit area. The highest LAI values were obtained from the silking to milk stage, and we found that the higher the plant density (i.e., the larger the number of plants per unit area), the larger was the LAI value (Fig 4). Siddique et al. [61] mention that the light interception rate of cereal crops tends to decline after flowering. Similarly, the photosynthetic activity of tissues declines as plants age, and the rate of dry matter accumulation is also reduced. Overall, regardless of treatment, we found in the present study that LAI values underwent a significant reduction after milk maturity, which affected the rates of light interception and dry matter accumulation. Yang et al. [17] showed that leaves are more sensitive to changes in solar radiation under low light conditions, and that low levels of solar radiation at high planting densities can accelerate leaf senescence. Consistently, in the present study, we recorded the highest LAI values in TNG1 and TNG7 plants subjected to the D4 treatment, and found that compared with the other treatments, these LAI values declined significantly after milk maturity (Fig 4). In this regard, Shibles and Weber [62] have pointed out that the rate of light interception by a crop canopy increases with an increase in LAI. Moreover, by altering the spatial arrangement of plants, Sarmento et al. [19] observed changes in the distribution of leaves, and found that a high plant density increased LAI and enhanced RUE and biomass conversion. In the present study, TNG1 and TNG7 plants subjected to the D4 treatment were characterized by the highest values of RUE, LAI, and aboveground dry matter. In the case of treatments D1 and D2 (in which we used the same planting densities), although there was no significant difference between the varieties with respect to RUE and LAI values, we did nonetheless note that TNG1 plants subject to treatment D2 had smaller LAI and RUE values than plants receiving the D1 treatment. Conversely, for TNG7 plants smaller LAI and RUE values were obtained for those plants subjected to treatment D1 (Table 4). Collectively, these finding thus indicate that within a reasonable range of planting densities, both RUE and LAI will increase with an increase in planting density. When the plant density is the same, RUE was affected by LAI. The RUE for field corn ranged between 2.10 g and 4.60 g MJ^-1^ and varied according to cultivar, planting density, and leaf structure.

### Limitation and uncertainty

Despite the multiple means of determining RUE the most common approach is that based on the slope of the relationship between dry matter production and cumulative intercepted photosynthetically active radiation (IPAR) [31–33]. However, in the present study, we were unable to estimate the extinction coefficient used in Beer’s Law, and accordingly, we chose to estimate RUE based on an approach using the relationship between the aboveground dry matter and IPAR, assuming that leaves completely intercept PAR as IPAR in the absence of any radiation loss and LAI effects, and that the ratio of IPAR to global solar radiation is 0.5 [29, 30]. During the course of the study, we assumed that complete interception of PAR as IPAR by the leaves in the absence of any radiation loss and LAI effects is in fact unlikely. IPAR is affected by crop leaf area and extinction coefficient, and we had concerns regarding the potential underestimation of RUE. It is thus suggested that given sufficient research funds and equipment. the Beer’s Law method should ideally be used for estimating IPAR, as this is likely to enhance the accuracy of RUE numerical estimations. Recording the dry matter weight and LAI during the entire growth period of crops provides researchers with a more comprehensive description of RUE. This approach does, nevertheless, necessitate considerable manpower and time to record data during the entire growth period. Moreover, unfavorable environmental conditions during the crop growing season will affect the RUE and dry matter of the crop. Despite these drawbacks, however, recording data throughout the entire growth period does provide valuable insights into the dynamic changes that crops undergo during cultivation.

In the present study, the D4 treatment was equivalent to a plant density of 140,000 plants per hectare, which is considerably higher than that used in previous studies [10, 45, 46]. However, we detected no reduction in dry matter attributable to the high plant density in our experiment. To discuss changes in dry matter at extreme planting densities, future studies need to conduct field trials using denser planting densities. Consequently, this would tend to indicate that within a reasonable planting density, selecting varieties with multiple excellent traits for planting can be conducive to obtaining higher dry matter yields.

## Conclusion

The optimum period for harvesting field corn is during the stage of physiological maturity when the aboveground dry matter has reached maximum levels. This association can be applied in the establishment of crop models, which can contribute to predicting the harvest period and the planning of effective field work schedules for different crops and varieties. In this context, it is generally acknowledged that solar radiation is an important factor influencing RUE, which is estimated based on the relationship between the accumulation of aboveground dry matter and IPAR, and can be used to illustrate the relationship between solar radiation and dry matter accumulation. The maize growth period is characterized by a number of discrete growth stages, which are affected to varying extents by meteorological condition, which if unfavorable, will result in a reduction of maize yields. Consequently, data obtained over the course of the entire growth period are of particular research value. Despite this importance, however, few published papers have assessed the relationships between the entire growth period of maize and climatic variables. It is hoped that in the future, there will be studies that assess detailed data collected over prolonged periods exceeding 10 years, together with important meteorological variables, such as accumulated temperature and radiation. Although in this study, we have focused on assessments of the aboveground dry matter of corn, which is part of the plant biomass, in future studies, it will be worthwhile examining the harvest index, the ratio of economic yield to biological yield, improvements of which would predictably increase economic yield. By enabling assessments of the dynamics of crop yield variation at an early stage of growth and improving reference values, this will be of particular utility to farmers. We can also anticipate more accurate measurements and new research fields in the routine surveying of corn fertility.

## Supporting information

S1 File Data of global solar radiation, LAI, and aboveground dry matter. (XLSX)
